# High-dose-rate brachytherapy with external beam radiotherapy versus low-dose-rate brachytherapy with or without external beam radiotherapy for clinically localized prostate cancer

**DOI:** 10.1038/s41598-021-85682-9

**Published:** 2021-03-17

**Authors:** Hideya Yamazaki, Koji Masui, Gen Suzuki, Norihiro Aibe, Daisuke Shimizu, Takuya Kimoto, Kei Yamada, Akihisa Ueno, Toru Matsugasumi, Yasuhiro Yamada, Takumi Shiraishi, Atsuko Fujihara, Koji Okihara, Ken Yoshida, Satoaki Nakamura

**Affiliations:** 1 Department of Radiology, Graduate School of Medical Science, Kyoto Prefectural University of Medicine, 465 Kajiicho Kawaramachi Hirokoji, Kamigyo-ku, Urology, 602-8566 Japan; 2 Graduate School of Medical Science, Urology, Kyoto Prefectural University of Medicine, 465 Kajiicho Kawaramachi Hirokoji, Kamigyo-ku, Kyoto, 602-8566 Japan; 3 Department of Radiology, Kansai Medical University, Hirakata, 573-1010 Japan

**Keywords:** Oncology, Urology

## Abstract

To compare the outcomes of localized prostate cancer treatment with high-dose-rate brachytherapy (HDR-BT) and low-dose-rate brachytherapy (LDR-BT), we examined 924 patients treated with HDR-BT + external beam radiotherapy (EBRT) and 500 patients treated with LDR-BT ± EBRT using multi-institutional retrospective data. The HDR-BT treated advanced disease with more hormonal therapy than LDR-BT. To reduce background selection bias, we performed inverse probability of treatment weighting (IPTW) analysis using propensity scores and excluded patients with T3b-4 disease/ initial prostate-specific antigen (PSA) levels > 50 ng/ml. The actuarial 5-year biochemical control rates (5y-bNED) were 96.3% and 95.7% in the HDR-BT and LDR-BT groups, respectively. The corresponding values were 100% and 96.5% in the low-risk group; 97.4% and 97.1% in the intermediate-risk group (97.2% and 97% in the higher titer group and 97.5% and 94.6% in the lower titer group, respectively); and 95.7% and 94.9% in the selected high-risk group, respectively. IPTW correction indicated no significant difference among the groups. The 5y-bNED in the HDR-BT + EBRT, LDR-BT + EBRT, and LDR-BT alone groups were 96.3%, 95.5%, and 97%, respectively (*P* = 0.3011). The corresponding values were 97.4%, 94.7%, and 96.6% (*P* = 0.1004) in the intermediate-risk group (97.5%, 100%, and 94.5% in the lower titer group [*P* = 0.122] and 97.2%, 96.2%, and 100% [*P* = 0.664] in the higher titer group, respectively) and 95.7%, 95.5%, and 100% (*P* = 0.859) in the high-risk group, respectively. The HDR-BT group showed a lower incidence of acute grade ≥ 2 genitourinary toxicities; the incidence of other early and late grade ≥ 2 toxicities were similar between the HDR-BT and LDR-BT groups. Acute genitourinary toxicity predicted the occurrence of late genitourinary toxicity. EBRT increased the risk of grade ≥ 2 gastrointestinal toxicity. HDR-BT + EBRT is a good alternative to LDR-BT ± EBRT for low-, intermediate-, and selected high-risk patients.

## Introduction

Although prostate cancer is a major malignancy in developed countries^1^, it is difficult to choose the best treatment option because there are many curative treatment options, such as surgery, external beam radiotherapy (EBRT), and brachytherapy (BT). BT is divided into permanent implant BT or low-dose-rate (LDR) BT and temporary implant BT or high-dose-rate (HDR) BT^1^. Compared to EBRT, BT delivers higher doses of radiation to the target lesion without excessive irradiation of the adjacent organs; therefore, it is considered to be one of the best radiotherapy options^2^. It can also ameliorate biochemical control as it delivers the highest biological equivalent dose compared to all other radiotherapy options.

LDR-BT is one of the standard treatment options for patients with low-risk prostate cancer^3^. LDR-BT was used as a boost to EBRT in patients with intermediate-to-high-risk disease, and outcomes were improved^1–3^. In the ASCENDE-RT trial, LDR-BT + EBRT led to improved biochemical control compared to EBRT (78 Gy)^4^. In contrast, the incidence of acute and late genitourinary (GU) toxicities was higher after LDR-BT boost, and there was a nonsignificant trend for worse gastrointestinal (GI) morbidity^5^. HDR-BT can also be used as a boost for EBRT (HDR-BT + EBRT) in patients with intermediate-^1,3^ and intermediate-to-high-risk prostate cancer^1^. In previous studies including ours, HDR-BT was used alone, and good efficacy was obtained in all risk groups^6,7^. We previously compared LDR-BT ± EBRT and HDR-BT monotherapy^7^, but we did not investigate the role of HDR-BT with EBRT. Therefore, in this study, we used freely available data regarding HDR-BT with EBRT^8^ to examine and compare the outcomes of HDR-BT with EBRT and LDR-BT ± EBRT. To reduce bias, we used an inverse probability of treatment weighting (IPTW) method using propensity scores. The aim of the present study was to compare the efficacy of HDR-BT with EBRT versus LDR-BT ± EBRT.

## Methods

### Patients

We examined the efficiency and toxicities of patients treated with HDR-BT (open data for public use)^8^ and LDR-BT in Kyoto Prefectural University of Medicine in retrospective fashion. Patient eligibility criteria included: treatment with HDR-BT with EBRT or LDR-BT ± EBRT, clinical TNM stage T1-T3 and N0M0 with histology-proven adenocarcinoma, availability and accessibility of data on pretreatment (initial PSA = iPSA) level, Gleason score sum (GS), T classification. The patients were staged according to the National Comprehensive Cancer Network (NCCN) 2015 risk classification as follows: low: T1–T2a, GS 2–6, and iPSA < 10 ng/mL; intermediate: T2b–T2c, GS 7, or PSA 10–20 ng/mL; and high: T3, GS 8–10, or PSA > 20 ng/mL^1^. PSA failure was defined using the Phoenix definition (nadir, + 2 ng/ml)^1^. Common Terminology Criteria for Adverse Events version 4.0 was used for toxicity analysis. All patients in LDR-BT group provided written informed consent and HDR-BT patients gave their informed consent during process of building public data. This study was conducted in accordance with the Declaration of Helsinki and with institutional review board permission (Kyoto Prefectural university of Medicine: ERB-C-1403).

### Treatment planning

#### LDR-BT with or without EBRT

**Figure 1:**
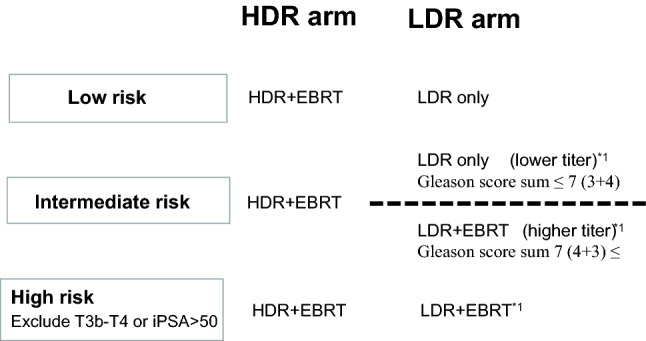
Patients selection criteria for Treatment scheme by risk factors. *1 = four patients received LDR + EBRT in lower titer of intermediate risk group, and seven patients received LDR only in higher titer of intermediate risk group, and two patients received LDR only in high group.

The implant technique was previously described in detail^7^. We performed permanent intraoperative Iodine-125 implantation (OncoSeed model 6711; General Electric Healthcare, Barrington, IL) using a modified peripheral loading method between 2005 and 2013. Inter-Plan version 3.4 (ELEKTA, Stockholm, Sweden) was used as the treatment planning system. We used combination therapy for T3 or Gleason score sum ≤ 8, or Gleason score sum 7 (4 + 3) cases (not for Gleason score sum 7 (3 + 4) cases)^9^ (Fig. 1). Our prescription dose for the clinical target volume (prostate) was 145 Gy (LDR-BT alone) or 110 Gy (LDR-BT with 40 Gy/ 20 fractions EBRT by three-dimensional conformal radiotherapy: 3D-CRT).

#### HDR-BT with EBRT

The multi-institution data was obtained from open data source^8^, and detailed method of the applicator implantation was described elsewhere^10^. All patients were treated with a combination of HDR and EBRT at various fractionations (Table 1). Of 1227 cases in open data source (6), we excluded (i) node positive case, (ii) metastasis case, and (iii) T3b ~ T4 or iPSA ≥ 50 ng/mL. Then 924 cases were obtained as HDR-BT group. The median dose of HDR used was 31.5 Gy (11–31.5 Gy) and that of EBRT was 39 Gy (39–51 Gy). The median fraction size of HDR was 9 Gy (6.3–11 Gy) and that of EBRT was 3 Gy (2–3 Gy). Patients who were administered EBRT comprised 909 (98.4%) on 3D-CRT and 15 (1.6%) on IMRT.

**Table 1:** Characteristics and treatment factors of patients.

Variables	Strata	HDR-BT n = 924			LDR-BT n = 500		*P* value
		No. or Median (range)	(%)		No. or Median (range)	(%)	
Age		71 (47–86)			69 (45–83)		**0.0029**
T category	1	228	(25%)		241	(48%)	** < 0.0001**
	2	379	(41%)		245	(49%)	
	3	317	(34%)		14	(3%)	
iPSA	ng/ml	12 (2.682–50)			7(1.4–46)		** < 0.0001**
Gleason score	-6	11	(1%)		284	(57%)	** < 0.0001**
	7	236	(26%)		193	(39%)	
	8-	382	(41%)		23	(5%)	
NCCN risk classification	Low	11	(1%)		200	(40%)	** < 0.0001**
	Intermediate	269	(29%)		259	(52%)	
	High	644	(70%)		41	(8%)	
Prescribed dose	11 Gy / 1fr + EBRT 45 Gy /15 fr or 51 Gy /17fr	145	(16%)	110 Gy + EBRT (40 Gy / 20fr)	69	(14%)	NA
	18 Gy / 2 fr + EBRT 39 Gy /13 fr or 51 Gy / 17fr or 48 Gy/16fr	233	(25%)	145 Gy	431	(86%)	
	20 Gy / 2fr + EBRT 30 Gy /15 fr or 46 Gy /23fr	13	(1%)				
	21 Gy / 3fr or 21 Gy /2 fr + EBRT 51 Gy / 17 fr or 45 Gy /15fr or 42 Gy/14fr	54	(6%)				
	25 Gy / 5fr + EBRT 51 Gy /17 fr	5	(1%)				
	31.5 Gy / 5fr + EBRT 30 Gy /10fr	468	(51%)				
Hormonal therapy	Yes	872	(94%)		399	(80%)	** < 0.0001**
Neoadjuvant	Months	10 (1–89)			6 (1–24)		
Adjuvant	Months	36 (1–93)			3 (1–19)		
	No	52	(6%)		101	(20%)	
Follow-up	Months	70 (2–177)			84 (17–148)		** < 0.0001**

*Bold values indicate statistically significance, NA; not available.

*HDR-BT* high dose rate brachytherapy, *LDR-BT* low-dose-rate brachytherapy, *EBRT* external beam radiotherapy (40 Gy/ 20 fractions).

### Statistical analysis

StatView 5.0 statistical software and R stat package^11^ were used for statistical analyses. R stat package was used only to calculate the propensity score and Inverse probability of treatment weighting (IPTW)^7,12^. Percentages were analyzed using chi-square tests and Student’s *t*-tests were used for normally distributed data. Mann–Whitney U-tests for skewed data were used to compare means or medians. The Kaplan–Meier method was used to analyze the biochemical control rate, survival, and accumulated toxicity and comparisons were made using log-rank tests. Cox’s proportional hazard model was used for uni- and multivariate analyses. *P* < 0.05 was considered statistically significant. Because the included patients were not randomized, unbalanced baseline characteristics could have led to selection bias and, hence, influence the decision to undergo LDR-BT or HDR-BT. The propensity score is defined here as the probability of being assigned to LDR-BT or HDR-BT given the patients characteristics. In the calculation of the propensity scores, the logistic regression model was used considering the baseline covariates shown in Table 2 (age, T classification, Gleason score, pretreatment PSA, and hormonal therapy). IPTW values were calculated from the propensity scores and represented the inverse probability of an HDR-BT group based on their characteristics. The treatment effects were recalculated using the IPTW with a Cox model. We weighted survival analysis using the inverse probability treatment weighting (IPTW) method, i. e., weighting patients who received LDR-BT by 1/propensity score, whereas patients who received HDR-BT were weighted by 1/(1–propensity score).

**Table 2:** Multi-variate analysis for biochemical control rate using Cox proportional hazards model.

Variable	Strata	Multivariate analysis		
		HR	95% CI	*P*
Age, years	≤ 74	1	(referent)	–
	75 ≤	1.275	0.656–2.479	0.4735
T classification	1	1	(referent)	–
	2	1.366	0.804–2.322	0.2488
	3	2.241	1.157–4.342	0.0168
Gleason score	≤ 6	1	(referent)	-
	7	1.088	0.615–1.925	0.7711
	8 ≤	1.655	0.842–3.254	0.142
Pretreatment PSA (ng/mL)	< 10	1	(referent)	–
	10 ≤ 20	1.161	0.689–1.955	0.5753
	20 <	1.357	0.716–2.572	0.3489
Hormonal therapy	No	1	(referent)	–
	Yes	0.882	0.450–1.730	0.7147
Treatment modalities	LDR-BT	1	(referent)	–
	HDR-BT	0.395	0.198–0.788	**0.0084**

Bold values indicate statistically significance.

*CI* confidence interval, *HR* hazard ratio, *NA* not available, *HDR-BT* high dose rate brachytherapy, *LDR-BT* low-dose-rate brachytherapy.

## Results

### Patient and tumor characteristics

The 1,424 patients with stage T1–T3 N0M0 prostate cancer were treated using HDR-BT with EBRT (n = 924) or LDR-BT (n = 500; treatment duration 2005–2013). The median patient age was 70 (range, 45–86) years. The patients’ clinical characteristics are shown in Table 1.

The median follow-up duration for the entire cohort was 75 (range: 2–177) months, with a minimum of 2 years for surviving patients or until death. A comparison of the characteristics of the two treatment modalities is shown in Table I. HDR-BT was used to treat patients with advanced disease and hormonal therapy than that in the LDR-BT group.

### Biochemical control and overall, prostate cancer-specific, and metastasis-free survival

**Figure. 2:**
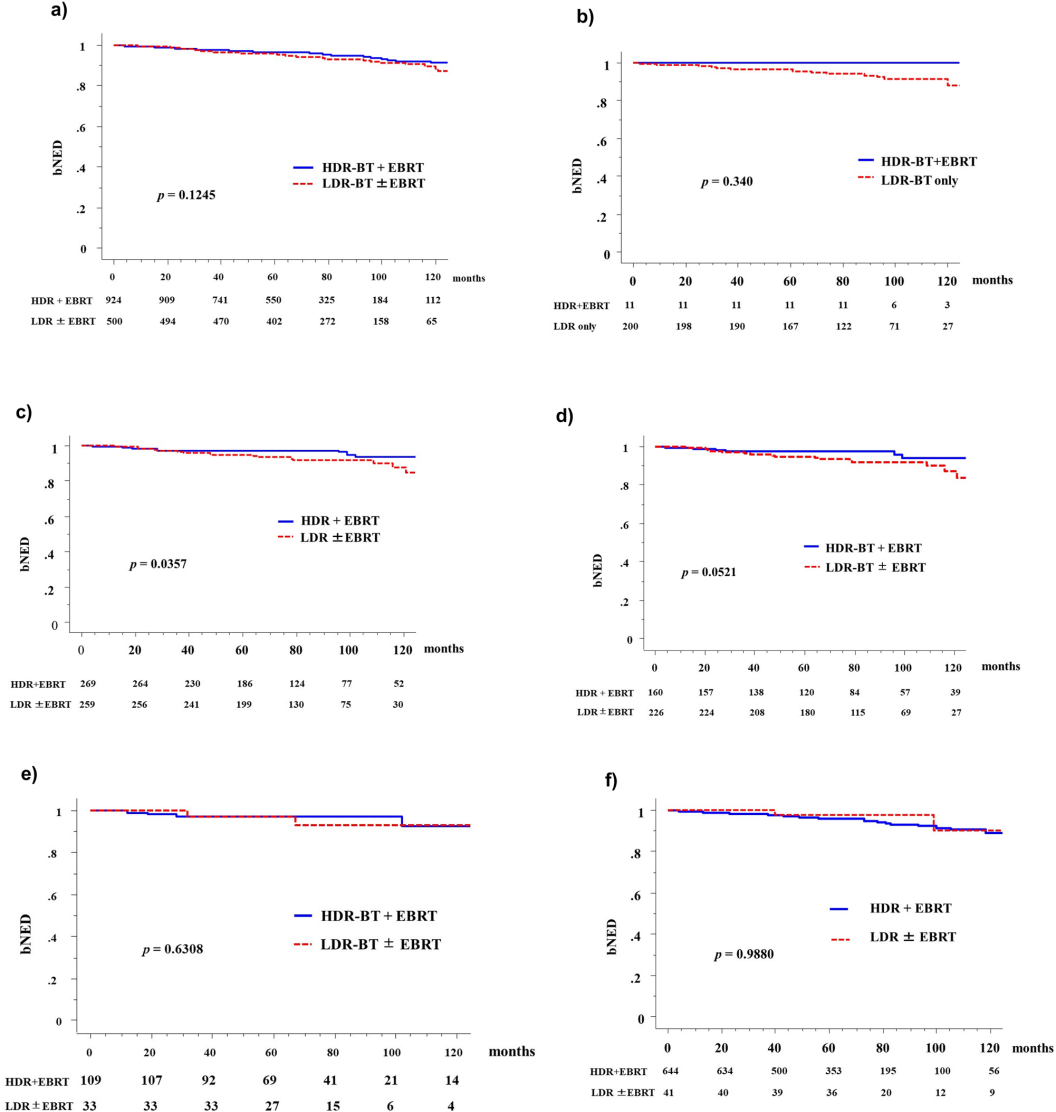
Biochemical control rates between HDR-BT with EBRT and LDR-BT with or without EBRT. (**a**) Biochemical control rates between HDR-BT with EBRT and LDR-BT with or without EBRT in the total population. (**b**) Low risk group. (**c**) Intermediate group. (**d**) Lower titer of intermediate group. (**e**) Higher titer of intermediate group. (**f**) High risk group. bNED = no biochemical evidence of disease.

The number of patients who showed biochemical failure was higher in the HDR-BT + EBRT group (44, 4.76%) than in the LDR-BT group (40 8.0%). The actuarial 5-year biochemical failure-free survival rates (biochemical disease-free survival = bNED) were 96.3% (95% confidence interval [CI]: 95.0–97.6%) and 95.7% (95% CI: 94.0–97.5%, *P* = 0.1214, Fig. 2; *P* = 0.070 after IPTW correction, Table 3) in the HDR-BT and LDR-BT groups, respectively. The corresponding values were 100% and 96.5% (*P* = 0.340 and 0.500, respectively, after IPTW correction) in the low-risk groups; 97.4% and 97.1% (*P* = 0.0357 and 0.700, respectively) in the intermediate-risk groups (97.2% and 97% [*P* = 0.6308] in the higher titer group and 97.5% and 94.6% [*P* = 0.0521] in the lower titer group, respectively); and 95.7% and 94.9% (*P* = 0.9880 and 0.700, respectively, after IPTW correction) in the selected high-risk group, respectively. IPTW correction indicated no significant difference in all groups (Table 3).

**Table 3:** The 5-year biochemical control rates corrected by Inverse probability of treatment weighting analysis.

Variable	Strata	PT No	HDR-BT	PT No	LDR-BT	Log-rank *P*—value	IPTW correction			
							Log-rank *P*-value	Cox *P*-value	HR	95%CI
NCCN risk classification	Low-risk	11	100.0%	200	96.5%	0.340	0.500	0.509	0.762	0.3399–1.707
	Intermediate-risk	269	97.4%	259	94.9%	0.036	0.700	0.657	1.041	0.8704–1.246
	Intermediate-risk Lower titer	160	0.975	229	0.947	0.0538	0.052	0.051	0.4097	0.1666–1.008
	Intermediate-risk Higher titer	109	0.972	30	0.966	0.5693	0.441	0.5339	0.5176	0.09676–2.768
	High-risk	644	95.7%	41	97.5%	0.988	0.700	0.748	1.052	0.7703–1.438
	Total	924	96.3%	500	95.7%	0.121	0.070	0.105	0.595	0.317–1.115

**Figure 3:**
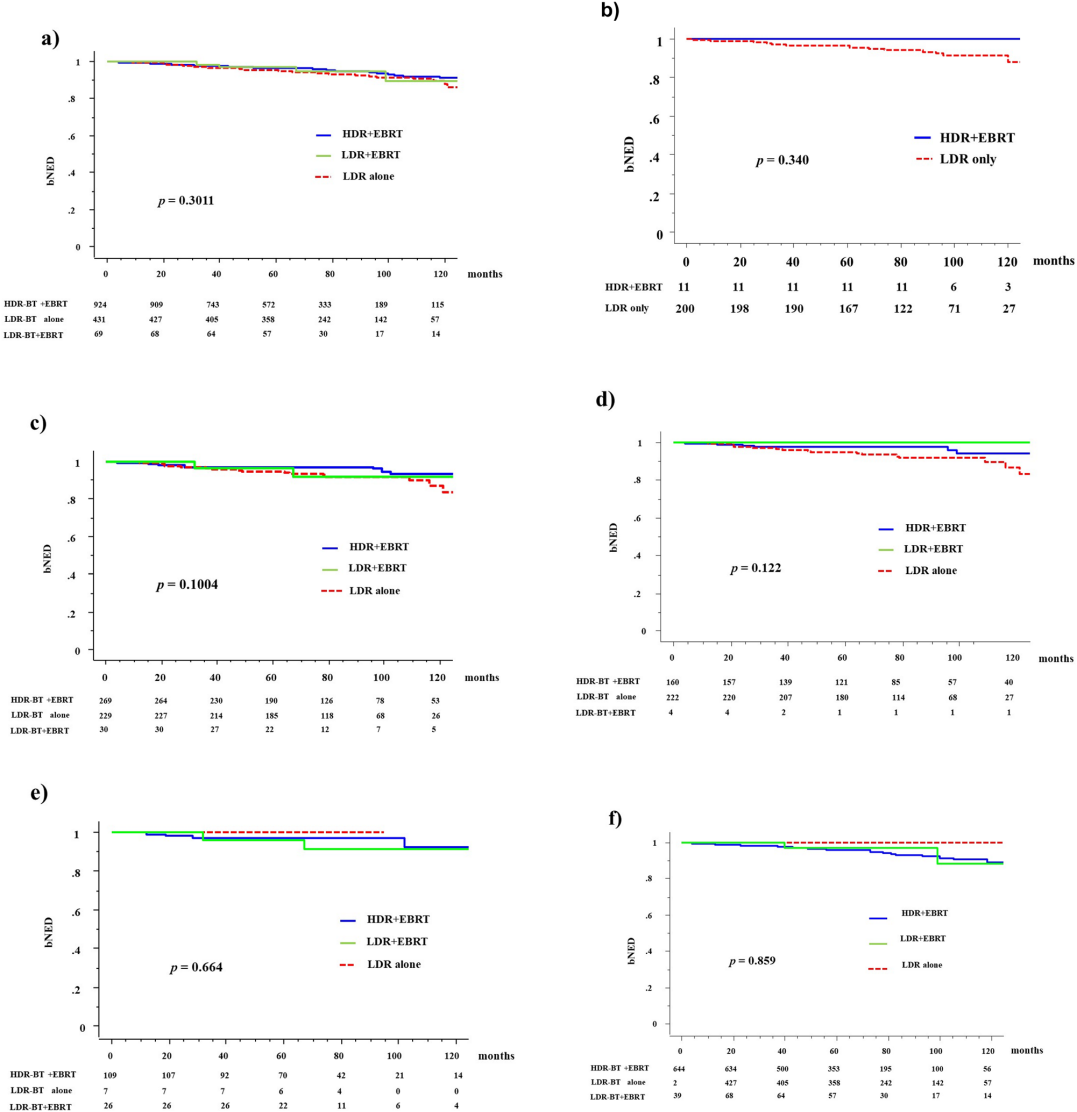
Biochemical control rates among three groups (HDR + EBRT vs. LDR + EBRT vs. LDR alone). (**a**) Biochemical control rates between HDR-BT with EBRT and LDR-BT with or without EBRT in the total population. (**b**) Low risk group. (**c**) Intermediate group. (**d**) Lower titer of intermediate group. (**e**) Higher titer of intermediate group. (**f**) High risk group. bNED = no biochemical evidence of disease.

The 5y-bNED in the HDR + EBRT, LDR + EBRT, and LDR alone groups were 96.3%, 95.5%, and 97%, respectively (*P* = 0.3011). The corresponding values were 97.4%, 94.7%, and 96.6% (*P* = 0.1004) in the intermediate-risk group (97.5%, 100%, and 94.5% in the lower titer group [*P* = 0.122] and 97.2%, 96.2%, and 100% [*P* = 0.664] in the higher titer group, respectively) and 95.7%, 95.5%, and 100% (*P* = 0.859) in the high-risk group, respectively (Fig. 3).

As shown in Table 2, the predictors of biochemical control on univariate analysis included treatment modality (LDR-BT vs. HDR-BT), T classification (T1 vs. T2 vs. T3), Gleason score (≤ 6 vs. 7 vs. ≥ 8), higher baseline PSA level (< 10 vs. 10–20 vs. < 20 ng/mL), and age (< 75 vs. ≥ 75 years). On multivariate Cox regression analysis, only a higher T category (T3) and treatment modality (HDR-BT better) remained significant for improving biochemical control. Table 4 shows the 5-year biochemical control rates according to the biological equivalent dose.

**Table 4:** The 5-year biochemical control rates according to biological equivalent dose (BED).

Schedule	BED (total)	Total		Low risk		Intermediate (lower titer)		Intermediate (higher titer)		High risk	
	(α/β = 1.5) (Gy)	PTNO (n)	5y-bNED (%)	PTNO (n)	5y-bNED (%)	PTNO (n)	5y-bNED (%)	PTNO (n)	5y-bNED (%)	PTNO (n)	5y-bNED (%)
HDR 11 Gy/ 1fr + EBRT 51 Gy /17fr	245	123	99.2			15	100	13	100	95	98.9
HDR 11 Gy/ 1fr + EBRT 45 Gy /15 fr	227	22	100			10	100	12	100		
HDR 18 Gy/ 2 fr + EBRT 39 Gy /13 fr	243	131	96.9	4	100	22	100	27	100	78	94.9
HDR 18 Gy/2fr + EBRT 48 Gy/16fr	270	1	100					1	100		
HDR 18 Gy/ 2 fr + EBRT51 Gy/ 17fr	279	107	93.3	3	100	30	93.2	8	100	66	92.2
HDR 20 Gy/ 2fr + EBRT 30 Gy/15 fr	223	1	100							1	100
HDR 20 Gy/ 2fr + EBRT 46 Gy/23fr	260	12	88.9			3	100	6	100	3	50
HDR 21 Gy/2 fr + EBRT 51 Gy/ 17 fr	321	1	100					1	100		
HDR 21 Gy/2fr + EBRT 42 Gy/14fr	294	2	100			2	100				
HDR 21 Gy/2 fr + EBRT 45 Gy/15fr	218	35	94.1	1	100	12	91.7	9	100	13	91.7
HDR 21 Gy/ 3fr + EBRT 51 Gy/ 17 fr	272	16	100	1	100	9	100	1	100	5	100
HDR 25 Gy/ 5fr + EBRT 51 Gy/17 fr	261	5	100					2	100	3	100
HDR 31.5 Gy/ 5fr + EBRT 30 Gy/10fr	253	468	96.1	2	100	57	98.2	29	89.5	380	96.2
LDR 145 Gy	154	431	95.5	200	96.5	222	94.5	7	100	2	100
LDR 110 Gy + EBRT 40 Gy/20fr	195	69	97			4	100	26	96.2	39	97.4

*bNED* biochemical control rate, *BED* = biological equivalent dose = nd(1 + d/[α/β]) : n = Number of treatment fractions : d = Dose per fraction in Gy.

**Figure 4:**
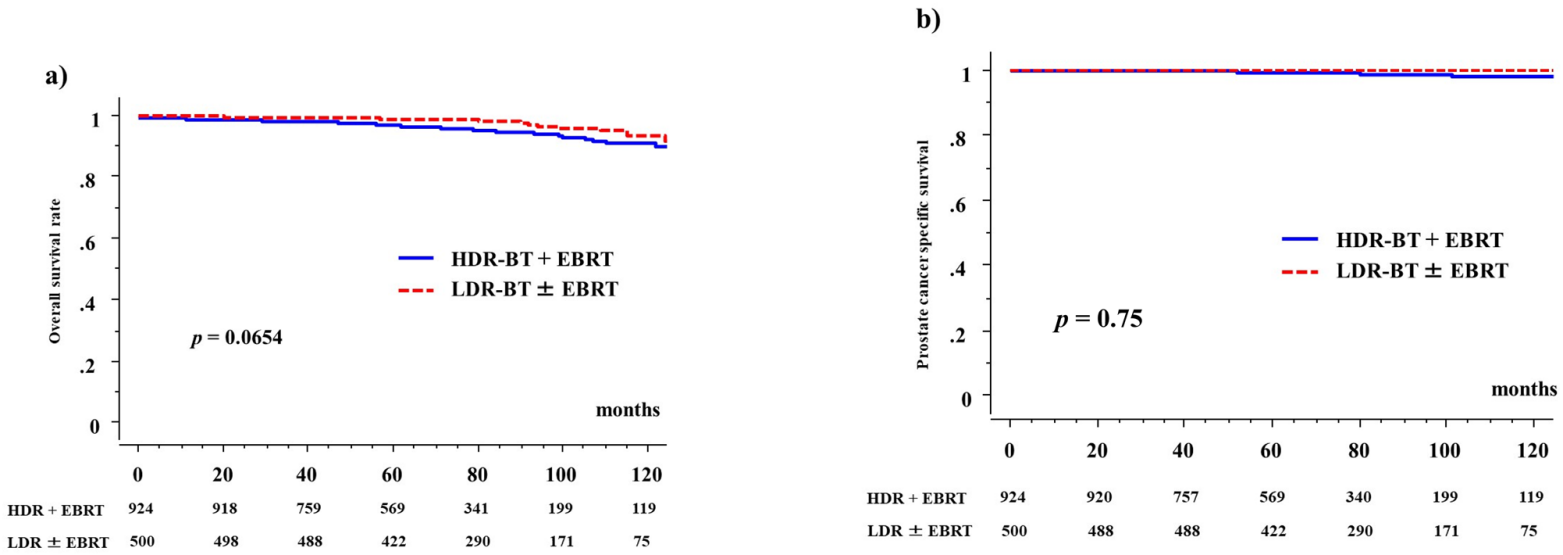
Overall and prostate cancer specific survival rate between HDR-BT with EBRT and LDR-BT with or without EBRT. (**a**) Overall survival rate. (**b**) Prostate cancer specific survival rate.

The 5-year overall survival rates were 97.4% (95% CI: 98.3–98.6%; 91.8% at 10 years) and 99% (95% CI: 98.1–99.9%, 93.6% at 10 years, *P* = 0.0654, Fig. 4) in the HDR-BT and LDR-BT groups, respectively. The 5-year overall survival rate was 100% in the low-risk groups, 98.1% (97.9% in the HDR-BT group and 98.4% in the LDR-BT group,* P* = 0.9331) in the intermediate-risk groups, and 97.2% (97.1 in the HDR-BT group and 97.6% in the LDR-BT group, *P* = 0.3399) in the selected high-risk groups. There were no significant differences in overall survival rates among the three risk groups (*P* = 0.0532).

There were nine prostate cancer-related deaths in this cohort (all high-risk patients who received HDR-BT with EBRT). The 5-year prostate cancer-specific survival rates were 99.5% (98.1% at 10 years) and 100% (100% at 10 years) in the HDR-BT and LDR-BT groups, respectively (*P* = 0.75, Fig. 4).

There were 23 cases of distant metastases (3, LDR-BT and 20, HDR-BT), and the 5-year distant metastasis-free survival rates were 98.2% (95.9% at 10 years) and 99.6% (98.6% at 10 year) in the HDR-BT and LDR-BT groups, respectively (*P* = 0.0702).

### Toxicity

#### Acute toxicity

The incidence of GI toxicity was similar in the HDR-BT with EBRT and LDR-BT ± EBRT groups (Table 5). The prevalence of GI toxicity was higher in the LDR-BT + EBRT group than in the LDR-BT alone group. However, grade 4 toxicities were not observed. The prevalence of acute GU toxicity was higher in the LDR-BT group than in the HDR-BT group. Grade ≥ 4 toxicities were not observed in both groups. The detailed acute toxicity profile (per event) is shown in Supplemental Table 1.

**Table 5:** Comparisons between HDR-BT and LDR-BT for toxicities.

Toxicities	Grade	HDR-BT		LDR-BT		*P*-value	LDR-BT alone		LDR-BT plus EBRT		*P*-value
		n = 924		n = 500			n = 431		n = 69		
		No	(%)	No	(%)		No	(%)	No	(%)	
(a) Acute toxicity											
Gastrointestinal	0	827	(90%)	436	(87%)	0.4588	388	(90%)	48	(70%)	* < 0.0001
	1	94	(10%)	62	(12%)		43	(10%)	19	(28%)	
	2	2	(0.2%)	2	(0.4%)		0	(0%)	2	(3%)	
	3	1	(0.1%)	0	(0%)		0	(0%)	0	(0%)	
Genitourinary	0	340	(37%)	37	(7%)	< 0.0001	33	(8%)	4	(6%)	0.8915
	1	499	(54%)	252	(50%)		218	(51%)	34	(49%)	
	2	82	(9%)	210	(42%)		179	(42%)	31	(45%)	
	3	3	(0%)	1	(0.2%)		1	(0%)	0	(0%)	

Toxicities	Grade	HDR-BT		LDR-BT		*P*-value	LDR-BT alone		LDR-BT plus EBRT		*P*-value
		n = 924		n = 500			n = 431		n = 69		
		No	(%)	No	(%)		No	(%)	No	(%)	
(b) Late toxicity											
Gastrointestinal	0	766	(83%)	446	(89%)	0.0142	396	(92%)	50	(72%)	* < 0.0001
	1	130	(14%)	46	(9%)		31	(7%)	16	(23%)	
	2	27	(3%)	8	(2%)		4	(1%)	3	(4%)	
	3	1	(0.1%)	0	(0%)		0	(0%)	0	(0%)	
Genitourinary	0	418	(45%)	202	(40%)	< 0.0001	176	(41%)	26	(38%)	0.7413
	1	361	(39%)	215	(43%)		182	(42%)	33	(49%)	
	2	87	(9%)	78	(16%)		68	(16%)	10	(15%)	
	3	58	(6%)	5	(1%)		5	(1%)	0	(0%)	

*HDR-BT* high dose rate brachytherapy, *LDR-BT* low-dose-rate brachytherapy, *EBRT* external beam radiotherapy.

*P value was calculated excluding columns of grade 3.

#### Late toxicity

Table 5b shows the incidence of late GI and GU toxicities. The incidence of GI toxicities was higher and that of GU toxicities was lower in the HDR-BT group than in the LDR-BT group. An elevated incidence of GI toxicities was observed in the LDR-BT + EBRT than LDR-BT (grade ≥ 2 toxicity: 1% vs. 4%, *P* < 0.0001); however, grade 3 toxicities were not observed.

**Figure 5:**
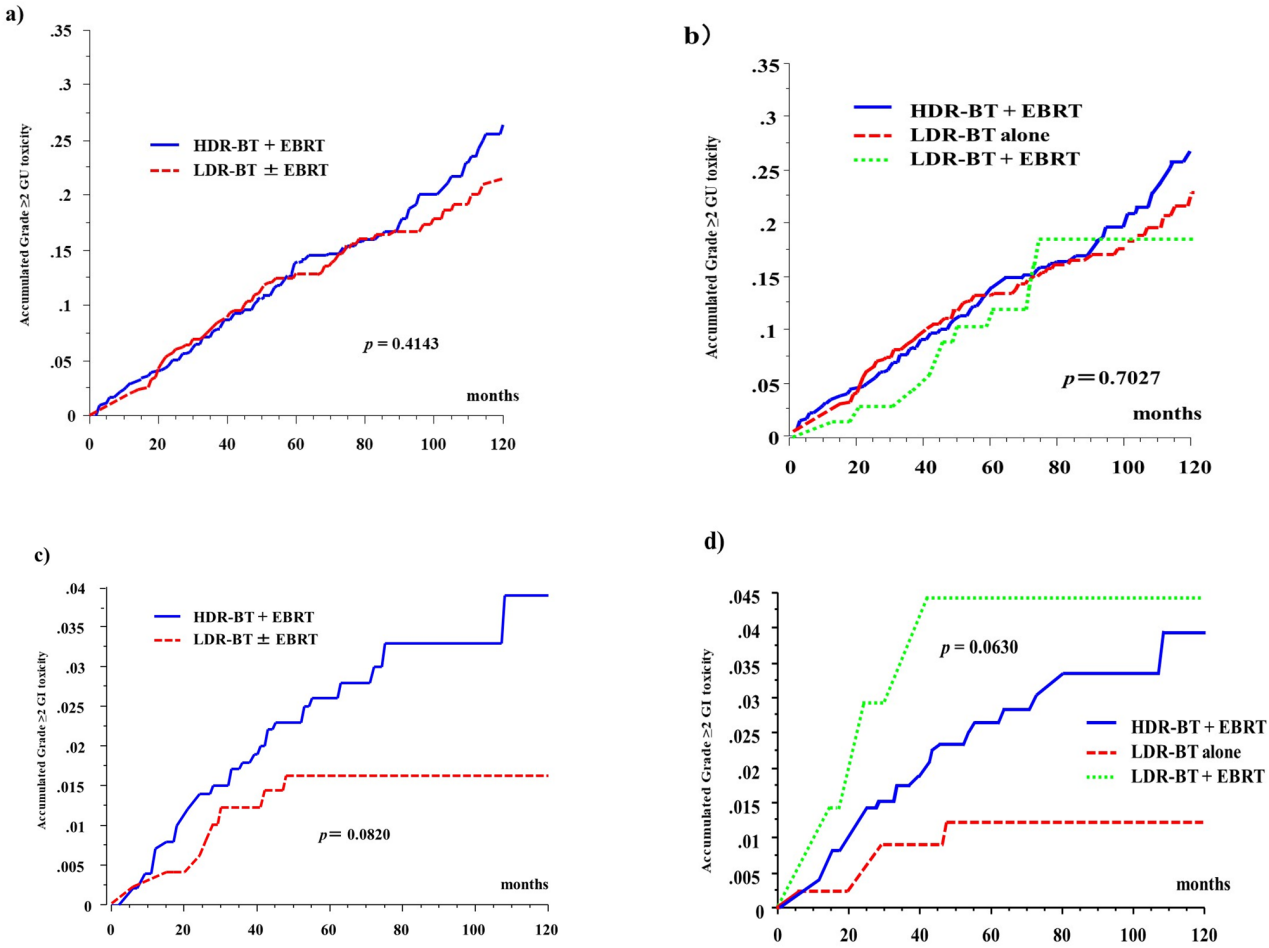
Accumulated incidence of grade ≥ 2 toxicity. (**a**) Accumulated incidence of grade ≥ 2 Genitourinary (GU) toxicity between HDR-BT with EBRT and LDR-BT with or without EBRT. (**b**) Accumulated incidence of grade ≥ 2 GU toxicity among three group. (HDR-BT with EBRT vs. LDR-BT alone vs. LDR-BT with EBRT). (**c**) Accumulated incidence of grade ≥ 2 Gastrointestinal (GI) toxicity between HDR-BT with EBRT and LDR-BT with or without EBRT. (**d**) Accumulated incidence of grade ≥ 2 GI toxicity among three group. (HDR-BT with EBRT vs. LDR-BT alone vs. LDR-BT with EBRT).

The 5-year cumulative incidence rate of grade ≥ 2 GU toxicities was 13.7% (26.5% at 10 years) in the HDR-BT group and 12.9% (22%) in the LDR-BT group (*P* = 0.4143; Fig. 5) and were 13.7% (26.5% at 10 years), 13% (22.5% at 10 years), and 12% (18.6% at 10 years) in the HDR-BT, LDR-BT alone, and LDR-BT + EBRT groups (*P* = 0.7027, Fig. 5), respectively.

The 5-year cumulative incidence rate of grade ≥ 2 GI toxicities was 2.6% (3.9% at 10 years) in the HDR-BT group and 1.6% (1.6% at 10 years) in the LDR-BT group (Fig. 5, *P* = 0.0820). The 5-year cumulative incidence rates of grade ≥ 2 late GI toxicities were 2.6% (3.9% at 10 years), 1.2% (1.2% at 10 years), and 4.4% (4.4% at 10 years) in the HDR-BT, LDR-BT, and LDR-boosted groups (*P* = 0.0630 among the three groups and *P* = 0.04 between the LDR-BT + EBRT group and LDR-BT only group, Fig. 5), respectively. Details of the late toxicity profile (per event) are shown in Supplemental Table 2.

Multivariate analyses revealed that the occurrence of acute grade ≥ 2 GU toxicities predicted the occurrence of late grade ≥ 2 GU toxicities (hazard ratio 2.745, *P* < 0.0001; Table 6). In addition, EBRT increased the probability of late GI toxicities (grade ≥ 2) in the LDR-BT group (LDR-BT + EBRT vs. LDR-BT alone, hazard ratio 8.239, *P* = 0.0123) and HDR-BT group (LDR-BT alone vs. HDR-BT + EBRT, hazard ratio 8.701, *P* = 0.0056).

**Table 6:** Multi-variate analysis of late grade ≥ 2 GI/ GU toxicity.

Variable	Strata	GI toxicity grade ≥ 2			GU toxicity grade ≥ 2		
		HR	95% CI	*P-*value	HR	95% CI	*P*-value
Age, years	< 75	1	(referent)	–	1	(referent)	–
	75 ≤	1.559	0.673–3.608	0.299	1.158	0.821–1.635	0.4036
NCCN	Low	1	(referent)	–	1	(referent)	–
Risk group category	Intermediate	0.826	0.190–3.601	0.7993	0.915	0.593–1.412	0.6881
	High	0.302	0.061–1.495	0.1423	0.761	0.450–1.288	0.3089
Hormonal therapy	No	1	(referent)	–	1	(referent)	
	Yes	0.816	0.253–2.634	0.7334	1.148	0.741–1.778	0.5361
Treatment modalities	LDR-BT	1	(referent)	–	0.966	0.489–1.904	0.9204
	LDR-BT + EBRT	8.239	1.851–36.667	**0.0123**	1	(referent)	–
	HDR-BT + EBRT	8.701	1.598–47.387	**0.0056**	1.724	0.882–3.369	0.1109
Acute toxicity	Grade 0–1	*NA			1	(referent)	–
	Grade 2–3				2.745	1.998–3.770	** < 0.0001**

Bold values indicate statistically significance.

*CI* confidence interval, *HR* hazard ratio, *NA* not available.

*Incidence of GI acute toxicity was too low to analysis.

## Discussion

Prostate BT (alone or combined with EBRT) has been proven to improve biochemical control; therefore, it is a standard treatment option for prostate cancer^1–3^. Using the best available statistical methods, this study showed equivalent outcomes in terms of bNED and overall and prostate cancer-specific survival among prostate cancer patients with varying risks (low, intermediate, and selected high risks) who received HDR-BT with EBRT and LDR-BT ± EBRT.

LDR-BT is one of the standard therapies for low-to-favorable intermediate-risk localized prostate cancer^1^. LDR-BT can be used for advanced disease; however, the patient selection criteria for delivering an LDR-BT boost remain controversial, especially for unfavorable intermediate- and high-risk groups^2,3^. HDR-BT has several theoretical advantages over LDR-BT^13^: (i) better tumor coverage even for metastases outside the prostate without unnecessary irradiation of organs at risk by optimization even after implantation^6^; (ii) better radioprotection for patients and staff; and (iii) a low α/β ratio for prostate cancer (1.2–1.5), which implies a radiobiological advantage over larger fraction sizes. HDR-BT treated more advanced cases than LDR-BT in our cohort. Several retrospective studies^14–16^ and a few preliminary and premature prospective studies have reported benefits of HDR-BT^17–19^.

LDR-BT uses a longer duration for radiation dose delivery (over 6 months; the half-life of iodine-125 is 60 days) than HDR-BT, which takes 10–15 min. Therefore, LDR-BT may increase the risk of GU toxicity in the acute phase compared to HDR-BT due to a longer recovery period. Grills et al. reported that HDR-BT is associated with less GU toxicity than LDR-BT^20^. A Canadian study also reported fewer GU and GI toxicities in patients undergoing HDR-BT with EBRT than in patients undergoing LDR-BT with EBRT^11^. Based on those findings, phase III trials for favorable risk groups (NCT02692105) and unfavorable intermediate-risk and high-risk groups (NCT01936883) have been conducted^18^; these trials are ongoing.

Our data were in line with the results from previous studies. A higher rate of acute grade ≥ 2 GU toxicities was observed in the LDR-BT (42.2%) group than in the HDR-BT with EBRT group (9%, *P* < 0.0001). Increased urethral toxicity related to LDR-BT may be attributed to a dose-rate effect or may reflect the ability of HDR to spare the urethra. However, the 5-year cumulative incidence rates of grade ≥ 2 late GU toxicities were similar in the HDR-BT and LDR-BT groups (13.7% and 12.9%, respectively; *P* = 0.4143). Regarding later GU toxicity, grade 3 obstruction was more common in the HDR-BT group (6.1%, Supplemental Table 2) than in the LDR-BT group (0.6%). It would be interesting to know if the higher rate of grade 3 late GU obstruction in the HDR-BT with EBRT group was mainly due to simple strictures that were easily dilated; this is a problem related to the toxicity grading system. The occurrence of acute GU toxicity predicted the occurrence of late GU toxicity, and late GU toxicities occurred continuously even 10 years later. Late GU toxicities are the main obstacle to BT; therefore, attention and efforts should be concentrated on reducing GU toxicity. For GI toxicity, EBRT increased the incidence of GI toxicities in the LDR-BT group and HDR-BT with EBRT group, compared to that in the LDR alone group. To reduce GI toxicity, advanced technology, i.e., IMRT^22^ and a spacer insertion between the rectum and prostate (i.e., SpaceOAR hydrogel, Boston Scientific Co. MA, USA) is underway; these maneuvers could be fruitful^23^. In general, the results of this study show that most patients did not experience long-term treatment-related severe toxicity.

This study has several limitations. First, its retrospective nature, limited follow-up time, and small sample size may limit the application of its findings to the general population. Thus, a longer follow-up with a larger sample is needed for reaching concrete conclusions. Second, our IPTW analysis cannot replace a randomized controlled study design because it only depends on known confounders and ignores the unknown confounders. Third, the roles of BT in very high-risk cases (T3b disease and/or iPSA levels ≥ 50 ng/ml) need to be studied. Fourth, although dose volume analysis, such as the analysis of D90 or the dose to the urethra, bladder/neck, and rectum, could be fruitful, these data were not available in the public database. Therefore, those exploration is left for future studies. Fifth, modern radiotherapy practice has employed IMRT for many years and 3D-CRT has been associated with increased toxicity, toxicity profiles may be higher than expected today, this is especially important when hypo-fractionation is introduced. Sixth, as longer use of ADT in the HDR group could mask the radiotherapy efficacy, the follow-up period of 70 months may be short to fully assess the outcomes. Seventh, though using a free database is beneficial, retrospective databases may not record toxicity outcomes properly.

## Conclusions

This study shows that HDR-BT with EBRT is a good alternative to LDR-BT with or without EBRT for low-, intermediate-, and selected high-risk patients, with an equivalent efficacy. HDR-BT led to less acute GU toxicity and an equivalent cumulative incidence of late grade ≥ 2 GU toxicities. EBRT increased the occurrence of GI toxicities.

## Supplementary Information

Supplementary Information

## Data Availability

The data of HDR-BT for this manuscript can be obtained from the public data base^8^ and LDR-BT was can be obtained from the author upon reasonable request.

## References

[1] The National Comprehensive Cancer Network (NCCN), NCCN Clinical Practice Guidelines in Oncology. Prostate Cancer, 2015 version 4. https://www.nccn.org/store/login/login.aspx? ReturnURL=https://www.nccn.org/professionals/physician_gls/pdf/prostate.pdf. 10.6004/jnccn.2010.0012 20141676

[2] Chin J (2017). Brachytherapy for patients with prostate cancer: american society of clinical oncology/cancer care Ontario joint guideline update. J. Clin. Oncol..

[3] Bittner NH (2017). The American College of Radiology and the American Brachytherapy Society practice parameter for transperineal permanent brachytherapy of prostate cancer. Brachytherapy.

[4] Morris WJ (2017). Androgen suppression combined with elective nodal and dose escalated radiation therapy (the ASCENDE-RT Trial): an analysis of survival endpoints for a randomized trial comparing a low-dose-rate brachytherapy boost to a dose-escalated external beam boost for high- and intermediate-risk prostate cancer. Int. J. Radiat. Oncol. Biol. Phys..

[5] Rodda S (2017). ASCENDE-RT: an analysis of treatment-related morbidity for a randomized trial comparing a low-dose-rate brachytherapy boost with a dose-escalated external beam boost for high- and intermediate-risk prostate cancer. Int. J. Radiat. Oncol. Biol. Phys..

[6] Yoshioka Y (2000). High-dose-rate interstitial brachytherapy as a monotherapy for localized prostate cancer: treatment description and preliminary results of a phase I/II clinical trial. Int. J. Radiat. Oncol. Biol. Phys..

[7] Yamazaki H (2019). High-dose-rate brachytherapy monotherapy versus low-dose-rate brachytherapy with or without external beam radiotherapy for clinically localized prostate cancer. Radiother. Oncol..

[8] An open data of multicenter data collection: outcome of radiation therapy for prostate cancer to establish a prognostic prediction system by machine learning (B17–278) https://www.khp.kitasato-u.ac.jp/ska/radiotherapy/arcivements/#results

[9] Yamada Y (2015). Permanent prostate brachytherapy and short-term androgen deprivation for intermediate-risk prostate cancer in Japanese men: outcome and toxicity. Brachytherapy.

[10] Ishiyama H (2017). Nationwide multi-institutional retrospective analysis of high-dose-rate brachytherapy combined with external beam radiotherapy for localized prostate cancer: an Asian Prostate HDR-BT Consortium. Brachytherapy.

[11] R-project home page: https://www.r-project.org/. Accessed Feb 2, 2018

[12] Rosenbaum PR (1983). The central role of the propensity score in observational studies for causal effects. Biometrika.

[13] Major T (2017). Dosimetric comparison between treatment plans of patients treated with low-dose-rate vs. high-dose-rate interstitial prostate brachytherapy as monotherapy: Initial findings of a randomized clinical trial. Brachytherapy.

[14] Barnes J, Kennedy WR, Fischer-Valuck BW (2019). Treatment patterns of high-dose-rate and low-dose-rate brachytherapy as monotherapy for prostate cancer. J. Contemp. Brachytherapy.

[15] Morgan TM (2018). Brachytherapy for localized prostate cancer in the modern era: a comparison of patient-reported quality of life outcomes among different techniques. J. Contemp. Brachytherapy.

[16] King MT (2019). A comparative analysis of overall survival between high-dose-rate and low-dose-rate brachytherapy boosts for unfavorable-risk prostate cancer. Brachytherapy.

[17] Fischer-Valuck BW (2019). A brief review of low-dose rate (LDR) and high-dose rate (HDR) brachytherapy boost for high-risk prostate. Front. Oncol.

[18] Dess RT (2019). The current state of randomized clinical trial evidence for prostate brachytherapy. Urol. Oncol..

[19] Hathout L (2019). A phase 2 randomized pilot study comparing high-dose-rate brachytherapy and low-dose-rate brachytherapy as monotherapy in localized prostate cancer. Adv. Radiat. Oncol..

[20] Grills IS (2004). High dose rate brachytherapy as prostate cancer monotherapy reduces toxicity compared to low dose rate palladium seeds. J. Urol..

[21] Rose T (2015). QOL comparison of acute side effect from a high dose rate vs low dose rate brachytherapy boost combined with external beam radiotherapy. Brachytherapy.

[22] Yamazaki H (2014). Transitioning from conventional radiotherapy to intensity-modulated radiotherapy for localized prostate cancer: changing focus from rectal bleeding to detailed quality of life analysis. J. Radiat. Res..

[23] Hamstra DA (2017). Continued Benefit to Rectal Separation for Prostate Radiation Therapy: Final Results of a Phase III Trial. Int J. Radiat. Oncol. Biol. Phys..

